# Serological Tests for Syphilis
*The basis of a communication given to the Southmead Hospital Clinical meeting.


**Published:** 1966-04

**Authors:** H. R. Cayton

**Affiliations:** Director, Public Health Laboratory Service, Bristol


### SEROLOGICAL TESTS FOR SYPHILIS*

BY

H. R. CAYTON, (M.B., CH.B., M.C.PATH.
*Director, Public Health Laboratory Service, Bristol*)

### THE WASSERMANN REACTION (C.L.W.R.)

The original Wassermann test was a complement-fixation test which used as the antigen treponema from the liver of a syphilitic stillborn child (Bordet, Gengou, Wassermann, 1906). Although the true Wassermann test is no longer carried out it is not uncommon for the term to be used as a generic expression for any complement-fixation test using heart extract as the antigen. Although these lipoidal antigens are not absolutely specific or sensitive for syphilis the tests are not difficult to perform and are widely available. The results are generally significant, but as long ago as 1910 it was recognized that these tests sometimes gave positive results with the sera of patients in whom there was neither history nor sign of luetic infection. At that time these occasional "false positives" were not a problem, as the tests were applied only to those in whom syphilitic infection was likely or could be expected. The population now being subjected to the tests is however, both quantitatively and qualitatively different, the great majority being ordinary persons, such as pregnant mothers, blood donors or emigrants, in whom syphilis is rare. In these groups it is crucial to recognize the "biological false positive" reaction for what it is, and to establish the status of the true positive reaction.

### FALSE POSITIVE REACTIONS

A "biological false positive" (B.F.P.) may be defined as a positive reaction, not due to a technical error, given by a serological test for syphilis using a lipoidal antigen, with the serum of a patient not infected with a pathogenic treponeme; that is syphilis, yaws, bejel and pinta have been excluded on clinical grounds.

The "acute" response appears soon after the onset of an acute infection and disappears quite quickly, usually within a few weeks and certainly in six months. An example is infectious mononucleosis, which condition may not only simulate secondary syphilis but also give rise to extremely high titres. The association of biological false positives with vaccinia should also not be forgotten, especially since vaccination against smallpox is often related in time with a request for a Wassermann test. Contrarywise the history of recent vaccination must not be allowed to serve as an excuse for failure to investigate further an unexpected or surprising result. The acute (B.F.P.) reactions are usually "weakly positive" or "doubtful" and discrepancies between different tests are common.

The chronic (B.F.P.) reactions persist for months or years. No known cause may be found but malaria and leprosy are recognized associations. Many persons with such reactions develop disseminated lupus erythematosus years later.

### THE "T.P.I." TEST

The "sensitivity" of a test is a measure of its ability to detect a syphilitic serum. The "specificity" of a test refers to its ability to give a negative response in the absence of syphilis. Different tests and different antigens vary in sensitivity and specificity. A

* The basis of a communication given to the Southmead Hospital Clinical meeting.

test with a 99 per cent specificity gives such a high proportion of biological false positive reactions that it is unacceptable. As defined, the test would record 990 of 1,000 negative sera correctly. Ten non-syphilitic sera would be "positive". In a collection of 1,003 sera three syphilitic sera may be expected, giving "true" positive reactions. It follows that with a test of this specificity only three of thirteen positive reactions is likely to be due to syphilis. Similarly with a test which has a specificity as high as 99·9 per cent, one in four of the positive reactions is likely to be false.

In the search for more specific tests, development have been directed to the use of virulent *Treponema pallidum* as the antigen. In 1949 Nelson and Mayer published a report on the phenomenon of *T. pallidum immobilization* (T.P.I.). This employs as the antigen *T. pallidum* obtained from inoculated rabbit testes. In the presence of complement and specific antibody, the motile treponemes are immobilized. The test is highly specific but is less sensitive than the lipoidal antigen tests. Immobilizing antibody develops later than the reagin detected by lipoidal antigen tests but once developed it persists for many years and possibly for life.

The indications for the T.P.I. test are:—

1. To distinguish between specific and non-specific positive reactions to lipoidal antigen tests;

2. The further investigation of patients suspected of syphilis whose lipoidal antigen tests are negative or equivocal.

The test is both difficult to perform and expensive. The serum must be sterile and free from antibacterial agents such as penicillin. The test does not distinguish between syphilis and other treponematoses.

The Fluorescent Treponemal Antibody test is simpler to perform and likely to become generally available as an acceptable alternative.

### THE REITER PROTEIN COMPLEMENT FIXATION TEST. (R.P.C.F.T.)

Complement fixation tests using cultivable treponemes (Reiter) as the source of antigen have been developed. Of the lipoid, polysaccharide, and protein which can be isolated from the organisms the protein fraction appears to be a group antigen shared with the virulent *T. pallidum.* It has been found that the R.P.C.F.T. has a very high specificity. In routine practice the reagents are deliberately adjusted to give maximum sensitivity. The test detects an antibody distinct from Immobilizing Antibody and the Wassermann reagin. In treated syphilitics Reiter Protein C.F.T. is less sensitive than the cardiolipin W.R. Occassionally the cardiolipin W.R. is negative and the Reiter protein W.R. positive; this is a finding not uncommon in neurosyphilis. When one or more reagin test (C.L.W.R.) is positive and the R.P.C.T. is negative the diagnosis is likely to be (a) treated syphilis, or (b) a biological false positive reaction. In the absence therefore of a definite history of treatment recourse to the T.P.I. and the Fluorescent Antibody test is essential.

In terms of practical serology it is now possible to base the diagnosis of syphilis on the detection of three distinct antibodies: W.R. reagin, Reiter protein antibody, and Treponema Immobilizing antibody; if all three tests are positive the diagnosis is virtually beyond doubt. In practice a combination of a positive lipoidal antigen test and a positive Reiter protein C.F.T. makes it extremely unlikely that the findings are non-specific.

### PRICE'S PRECIPITATION TEST (P.P.R.)

In addition to the two complement fixation tests (C.L.W.R., and R.P.C.F.T.) the Bristol laboratory includes in its normal "battery" a flocculation test (Price's precipitation test, P.P.R.). This provides a reproducible test which can be used quantitatively in the control of treatment. This test is very specific but undersensitive. Patients become very depressed if their serum tests fail to show a steady progress to reversal. The technical process of the P.P.R. is so different from that of complement fixation that the routine performance of the test in parallel provides a valuable technical check on the handling of the specimens.

### CONCLUSIONS

In testing sera from patients whose symptoms and signs might be syphilitic in origin, sensitivity is of primary importance. The routine tests applied to the sera from normal populations must be highly *specific*, but these tests (T.P.I.) are difficult and cannot be applied to all sera. The routine service can only 'screen' thousands of sera with the object of selecting those requiring more intensive investigation. Clinical information is essential for a rational interpretation of the results; if none is available comment may be properly withheld.

Syphilis is now uncommon and the general use of antibiotics for intercurrent non-specific disease can mask or suppress its early signs. The syphilitic origin of later manifestations may be quite unsuspected. Patients with positive serological reactions require a full investigation and the serological results must be confirmed. When the diagnosis is established, contacts must be sought out and examined, not forgetting that there must be at least one infected, and possibly infective, contact outside the family. The incidence of syphilis is increasing again, and rose by 22½ per cent in 1964 as compared with 1963. Only by serology can latent syphilis be recognized. Once the disease is recognized the rewards of successful treatment are great, rewards which in terms of prevented suffering and averted disability are wholly satisfying to all concerned.

### REFERENCES

Sequeira, P. J. (1962). *Brit. J. of Venereal Diseases* **38,** 9–18.

U.S. Public Health Service (1961). *Syphilis. Modern Diagnosis and Management.* Publication No. 743, Washington, U.S. Govt. Printing Office.

